# Supplementary material for: Awareness of anaphylaxis among public in Al-Ahsa City of Saudi Arabia: A cross-sectional study
Source: PLoS One. 2024 Dec 30;19(12):e0315722. doi: 10.1371/journal.pone.0315722 (PMC11684614; doi:10.1371/journal.pone.0315722)
Supplement: Attachment — Submitted filename: Anaphla report.docx [file pone.0315722.s001.docx]

**Reviewer report for PLOS one Journal**

Title: Awareness of Anaphylaxis Among Public in Al-Ahsa City of Saudi Arabia: A Cross-sectional Study

1. **Abstract:**
   1.1. The result section in abstract: the author should put P-value to confirm the significance at the end of the sentence (Older age, more education, and Job status were associated with higher scores.
2. **Introduction:** suitable and expressive.

The objectives are clear, and so the presentation

3. **Methods:** the methods are well described. The author should mention the statistical tests which were used for the data analysis.
4. **Results section:**

In this part:

4.1. I suggest the authors should define EpiPen for anaphylaxis before the knowledge of using it.
4.2. The authors did not make comparisons of the knowledge score among the participants such as males and females, marital status, education levels, and so on
4.3. The authors did mention the factors affecting the Knowledge, Attitude regarding the awareness of Anaphylaxis.

**I suggest the followings:**

4.4. The authors should obtain Odd ratios for the factors affecting knowledge of Anaphylaxis among the participants using regression test
     4.4.1. The authors should add a regression table to demonstrate the factors that influence the Knowledge or awareness of Anaphylaxis

5. **Discussion:**  satisfied
6. **Conclusion:** satisfied
