# Supplementary material for: Awareness of anaphylaxis among public in Al-Ahsa City of Saudi Arabia: A cross-sectional study
Source: PLoS One. 2024 Dec 30;19(12):e0315722. doi: 10.1371/journal.pone.0315722 (PMC11684614; doi:10.1371/journal.pone.0315722)
Supplement: Attachment — Submitted filename: Letter of Response.docx [file pone.0315722.s002.docx]

Dear Editor

We sincerely thank you and the reviewers for their thoughtful and constructive feedback on our manuscript titled "*Awareness of Anaphylaxis Among Public in Al-Ahsa City of Saudi Arabia: A Cross-sectional Study*." Below, we provide detailed responses to each of the reviewer’s comments and outline the revisions made to the manuscript.

**Reviewer Comments and Responses**

**4.1 Suggestion:** The authors should define EpiPen for anaphylaxis before discussing participants' knowledge of its use.
**Response:** We have modified the manuscript to include a definition of EpiPen in the results section prior to addressing participants’ knowledge of its use, as suggested.

**4.2 Suggestion:** The authors did not make comparisons of the knowledge score among participants, such as males and females, marital status, education levels, etc.
**Response:** The difference in mean knowledge scores of anaphylaxis among participants (e.g., males and females, marital status, education levels) has been analyzed and represented in **Table 1: Demographic Profiles.**

**4.3 Suggestion:** The authors did mention the factors affecting the knowledge and attitudes regarding awareness of anaphylaxis. The authors should obtain odds ratios for these factors using a regression test.
**Response:**

- While the primary aim of this study is to assess public awareness of anaphylaxis in the Kingdom of Saudi Arabia (KSA) and calculate the mean awareness score based on survey responses, the secondary objectives do not include identifying factors influencing participants’ knowledge.
- The statistical methods employed were selected based on the nature of the variables. Specifically:
  - **Chi-square tests** were used for bivariate analyses to explore associations between variables.
  - For variables with more than two categories, **Analysis of Variance (ANOVA)** was applied.
  - For variables with only two categories, **independent t-tests** were employed.
- Conducting regression analysis and presenting a regression table to identify factors influencing knowledge is outside the approved objectives and scope of this study.

**4.4 Suggestion:** The authors should add a regression table to demonstrate the factors that influence knowledge or awareness of anaphylaxis.
**Response:** The primary aim and statistical framework of this study were focused on descriptive and comparative analyses of awareness scores. Adding regression analyses, including odds ratios and regression tables, would require expanding the study's objectives and revising the methodology. As this falls beyond the scope of the approved research, we respectfully chose not to include regression analyses in the current manuscript.

We hope these responses and revisions address the reviewers' concerns adequately. We are grateful for the opportunity to improve our manuscript and remain available for any further clarifications or additional modifications.

Thank you for your consideration.

Sincerely,

Dr.Ahmed Alanazy
